# Demographic and Clinical Determinants of Tuberculosis and TB Recurrence: A Double-Edged Retrospective Study from Pakistan

**DOI:** 10.1155/2022/4408306

**Published:** 2022-11-28

**Authors:** Mariam Ahmed Mujtaba, Matthew Richardson, Hira Shahzad, Muhammad Ishaq Javed, Ghazala Kaukab Raja, Pakeeza Arzoo Shaiq, Pranabashis Haldar, Sadia Saeed

**Affiliations:** ^1^University Institute of Biochemistry and Biotechnology, Pir Mehr Ali Shah Arid Agriculture University, Rawalpindi 46000, Pakistan; ^2^NIHR Leicester Biomedical Research Centre, Department of Respiratory Sciences, University of Leicester, Leicester, UK

## Abstract

**Objective:**

TB recurrence is the second episode of TB after initial treatment bringing about an additional 7% load in TB burden intensified by 17.7% of multidrug-resistant recurrent cases. It is necessary to curb recurrence so that attempts to deal with active disease can be made more effective. This study aimed to characterize sociodemographic and clinical factors associated with recurrent TB in a high-burden setting. *Methodology*. A retrospective case-control study was carried out at two hospitals in Rawalpindi, Pakistan. TB patients and controls were included in the study. Sociodemographic and clinical data were collected by questionnaire from all subjects. Multivariate regression analysis was performed to determine factors associated with TB and TB recurrence respectively.

**Results:**

In our study cohort, factors significantly associated with TB were low BMI (OR: 0.961 (CI 0.954–0.968), *p* < 0.001), female gender (OR: 2.065 (CI 1.922–2.219), *p* < 0.001), being single/unmarried (OR: 1.214 (CI 1.109–1.328), *p*=0.003), middle-income status (OR: 1.935 (CI 1.616–2.323), *p* < 0.001), smoking (OR: 1.567 (CI 1.435–1.710), *p* < 0.001), and diabetes mellitus (OR: 1.142 (CI 1.017–1.278), *p*=0.023). TB recurrence constituted 11.2% of patients presenting to the hospital. Compared with the first episode of TB, cases with recurrence were more likely to be older (OR: 1.011 (CI 1.004–1.017), *p* < 0.001), have disease awareness (OR: 1.906 (CI 1.486–2.437), *p* < 0.001), smear positive (OR: 2.384 (CI 1.650–3.536), *p* < 0.001), and be drug-resistant (OR: 5.615 (CI 4.265–7.386), *p* < 0.001).

**Conclusion:**

In the present study cohort, low BMI, female gender, being single, middle-income status, being unemployed, smoking, and being diabetic came out to be the sociodemographic and clinical risk factors for TB. Further exploring the TB cases increasing age, drug resistance and smear positivity stood out to be the major sociodemographic and clinical factors of TB recurrence despite high disease awareness.

## 1. Introduction

Tuberculosis (TB) is an airborne infectious disease, caused by *Mycobacterium tuberculosis (Mtb)*, that is still affecting the world despite global efforts and funding to eradicate it. According to the Global Tuberculosis Report of 2020, over 10.0 million people were affected and 1.4 million people died in 2019 [1, 2]. It is representing only a 9% reduction in TB incidence from 2015 to 2019 and is well below the target reduction of 20% needed to be on track with the World Health Organization (WHO) END-TB strategy [1]. This slow decline in TB reduction rate underlines the global need for additional effective TB-control measures. Globally, an additional 7% of the TB burden is caused by TB recurrence in previously treated individuals [1] and this number is significantly higher in high-incidence countries [3–5]. Drug resistance is another tier of disease burden with a prevalence rate of 17.7% in relapse cases as compared to 3.3% in new cases [1].

Recurrence of TB can occur because of a relapse of a partially treated previous infection or can be reinfection that stems from a new strain of *Mtb* [6]. Reinfection is probably more prevalent in countries with high TB incidence and HIV (human immunodeficiency virus) infection, where re-exposure risk and host susceptibility are respectively increased [7–9]. Relapse of TB is the more likely cause when recurrence presents within 12 months of completing a course of TB treatment [10]. Apart from clinical presentation, sociodemographic factors play a key role in the spread of TB disease as TB is epitomized by “poverty, stigmatization, economic distress, marginalization, and vulnerability” [1]. Various factors like age [11], gender [11–13], positive sputum culture [13], co-morbidities like HIV [4, 13–21], diabetes mellitus [11], multidrug-resistant tuberculosis (MDR-TB) [11, 22], and low adherence to treatment during the initial TB event [13, 14, 23] have been reported as risk factors of TB recurrence.

Pakistan is a TB endemic country with a disease burden of 5.7% [1], accounting for 61% of the burden in WHO's Eastern Mediterranean Region [24]. There is no country-level report on the recurrence rate from Pakistan; however, recently, a hospital-based study from Rawalpindi, Pakistan, reported 5.83% of prevalent pulmonary TB cases to have the recurrent disease [25]. In a resource-restricted setting like Pakistan where molecular genotyping for strain identification and follow-up study needs additional funding sources investigating recurrence rate and social determinants of TB and TB recurrence can facilitate TB case reporting. To investigate this, we performed a retrospective case-control study at two large hospitals in Rawalpindi, a commercial and industrial city in Pakistan, with demographic characteristics representative of the nation.

## 2. Methods

A retrospective case-control study involving 3999 subjects was conducted between May, 2018, and September, 2019, to explore sociodemographic and clinical aspects of TB and its recurrence in Pakistan. The study was approved by the ethics committee of Pir Mehr Ali Shah Arid Agriculture University, Rawalpindi, as well as by the ethics committee of Federal Government Tuberculosis Hospital Asghar Mall (Approval number: E-7(4)/2012-TBC/Training) involving human subjects. Data were collected through a designed questionnaire and informed written consent was taken from all subjects visiting the outpatient departments (OPDs) of Leprosy Hospital, Rawalpindi, and Federal Government TB Hospital, Rawalpindi. Both these hospitals are reference centers for patients of Rawalpindi-Islamabad. Among total subjects, 2000 were TB cases defined by evidence of abnormalities on chest X-ray compatible with TB, bacterially confirmed (sputum positive), and taking anti-TB medication. In some cases, along with chest X-ray presence of *Mtb* was detected with Xpert Mtb-RIF (Cepheid Inc). The remaining 1999 subjects were considered controls who visited hospitals either due to any other illness or are household contacts of TB patients who also underwent TB diagnosis and were excluded through chest X-ray.

Individuals of all ages (from <1 year to 92 years) were included in the study and no matching for age and gender was done. Among the sociodemographic factors, marital status, body mass index (BMI), educational status at three levels (uneducated, secondary level, and higher secondary level), socioeconomic status at three levels (low, i.e., PKR < 25000, middle, i.e., PKR 25001–40000, and high, i.e., PKR > 40000), provincial distribution (Punjabi and non-Punjabi), family history of disease, and employment status (employed and unemployed) were recorded. Among the other factors, Bacillus Calmette-Guerin (BCG) vaccination, HIV status, disease awareness, smoking, and co-morbidities like diabetes mellitus (DM) and cardiovascular diseases (CVDs), acid-fast bacillus (AFB) sputum test at three levels (nil, negative and positive), and drug resistance (drug-resistant and drug-susceptible) as well as for the presence of *Mtb* Xpert Mtb-RIF test (tested and not tested) were computed.

The data were analyzed using *R* software (R-4.1.1) [26]. The Shapiro–Wilk test was performed to assess for the distribution of continuous variables (age and BMI). The data were summarized by the descriptive statistics using frequency (percentage) of the sociodemographic and clinical factors and median and interquartile range of continuous variables. Univariate analysis was done using the Chi-square test (*p* < 0.05) for the association of sociodemographic and clinical factors with disease status as the dependent variable. Pearson correlation was used for measuring the association of factors with one another and only those variables with a correlation coefficient value of <0.6 were included in the multivariate model (Supplementary File (available here)). Finally, a multivariate logistic regression model was used, and the measure of association was odds ratio (OR) and 95% confidence intervals (95%CI). A *p* value of <0.05 was considered statistically significant.

## 3. Results

### 3.1. Sociodemographic and Clinical Characteristics of Patients with TB

The sociodemographic and clinical characteristics of both groups are summarized (Table 1). In univariate analyses, compared with controls attending our two hospitals, cases were younger (median age 30), had a lower BMI (median 18.5) comprised a higher proportion of males (60.4%), and a larger proportion was employed (53.4%). 75.1% of the cases belong to Punjab province, 87.2% have monthly income <25000, and 38.4% were uneducated. Of the clinical characteristics compared between the two groups, smoking was more prevalent in cases compared to controls. The HIV co-infection rate in our cohort was very low (<1% in both groups) and only one-quarter of patients had a history of BCG vaccination. The univariate analysis resulted in gender, occupation, provincial status, marital status, socioeconomic status, educational status, BCG vaccination, family history, smoking, and diabetes mellitus being associated with cases. Although statistically significant, variables like occupation and BCG vaccination were associated with gender and marital status (*r* ≥ 0.6), respectively. These two variables were excluded from multivariate analysis.

We then proceeded to perform a multivariate logistic regression analysis that is shown in Table 1. This analysis confirms the significance of female gender (OR: 2.065, CI 1.922–2.219), lower BMI (OR: 0.961, CI 0.954–0.968), being single (OR: 1.214, CI 1.109–1.328), having middle-income status (OR: 1.935, CI 1.616–2.323), smoking (OR: 1.567, CI 1.435–1.710), and diabetes mellitus (OR: 1.142, CI 1.017–1.278) as factors associated with cases. Among these variables, female gender, middle income, and smoking had the highest odds of developing TB.

### 3.2. TB Recurrence

To identify factors associated with TB recurrence, we compared patients with the second episode of TB with primary TB cases. There were 223 patients (11.2%) with recurrent TB in our cohort. The sociodemographic, clinical, and TB diagnostic characteristics of patients with primary and recurrent TB are given in Table 2. In univariate analyses, patients with recurrent TB were older (median 32), and a lower proportion had a history of BCG vaccination (19.7%). Important differences were observed in disease characteristics of patients with recurrent TB, with a higher proportion having pulmonary TB (91%), smear positivity in sputum (75.8%), and drug-resistant (39%). We also note that 81.2% of TB patients with recurrent TB have Xpert *Mtb*-RIF testing; however, this proportion was only 33.6% in patients with primary TB.

In the multivariate regression model, age, BMI, disease awareness, AFB sputum, and drug resistance were included. All of these were found to be significantly associated with recurrent TB (Table 2). The highest odds of developing TB recurrence were attained from drug resistance (OR: 5.615, CI 4.265–7.386) followed by smear positivity in sputum (OR: 2.384, CI 1.650–3.536), disease awareness (OR: 1.906, CI 1.486–2.437), and increasing age (OR: 1.011, CI 1.004–1.017).

## 4. Discussion

This large case-control study of patient-level data from two hospitals in Pakistan is representative of the wider community in the region and reflects the epidemiology of TB in this high-burden setting. We have investigated the sociodemographic and clinical characteristics of TB patients and identified that female gender, low BMI, middle-income status, single/unmarried individuals, and smoking as factors associated with TB cases. Further, we found that smear positivity, drug resistance, and increasing age are significantly associated with TB recurrence despite disease awareness in this population.

In our study, around 60% of TB patients were males similar to the 65% global prevalence of TB in males reported by the World Health Organization in 2016 [27]. Interestingly, the female gender has two-fold higher odds of developing TB compared to males in our analysis. Our results are consistent with many studies in different settings that reported a higher prevalence rate in men but an association with TB is higher in females [28–30]. This has been explained by sociocultural differences that place a lower priority on women's health and are not supposed to seek healthcare for themselves.

According to results of the present study, TB risk is 1.2 times higher in unmarried individuals compared to married individuals. In 2018, a systematic review of demographic factors conducted by Mohidem in Malaysia reported that single individuals are more likely to have TB [31]. Similarly, a case-control study in three countries of West Africa reported being single as a risk factor of TB [32]. Lack of emotional support and socioeconomic difficulties faced by unmarried individuals might be the potential reason for the higher risk of TB among unmarried persons.

Findings of the current study revealed association of low BMI with TB and are consistent with many studies in other high TB burden countries including South Africa [33], India [34], Sri Lanka [35], Korea [36], Taiwan [37, 38], and Colombia [39]. There is a reverse logarithmic relationship between BMI and TB. This relationship is attributable in part to weight loss before TB infection as evidenced by a recent study in 2020 that showed a 3-fold higher prevalence of underweights in TB than controls [40]. Body mass reflects adipose tissues and individuals with low visceral fat are vulnerable to TB because of a decrease in both pro-inflammatory cytokines like tumor necrosis factor (TNF) and a T-cell population that affects adaptive immune response by T cells in TB [41, 42].

We found a medium-income group represented by a monthly income of PKR. 25001–40000 (i.e., USD 123–197) have a 1.9 times higher risk of developing TB compared to those earning more than PKR. 40000. This is indicative of a low socioeconomic class as this income is a source of livelihood both for the earner as well as dependents. Socioeconomic life affects lifestyle by changing the priorities such as care seeking, nutritional status, and living conditions. Low socioeconomic status and poverty are important indicators of TB documented in many studies in different settings with increasing poverty increasing the risk of TB [43–46].

We investigated that there is a higher risk of TB in smokers compared to nonsmokers as documented in many previous studies [47–51]. Smoking promotes the adherence of bacteria by changing the mucociliary function of bronchi and exacerbating pulmonary inflammation and oxidative stress which contributes to increased lung damage and cavitation with pulmonary TB and in some studies is reported to increase the risk of TB recurrence [52].

Diabetes mellitus (DM) is another risk factor for tuberculosis in our cohort like several previous studies [53–55]. This is because DM alters the immune response in the host increasing the chance of TB infection [55, 56]. DM decreases the IFN-*γ* level [57, 58] as well as the antigen-presenting ability of macrophages. IL-8 and IL-22 drop in case of TB-DM co-disease state affecting phagocytosis and TB clearance [59].

The rate of recurrence diagnosed in our study was 11.2%, which is higher than the previous report (5.83%) from Pakistan published in 2021 [25] and studies from other regions of the globe, i.e., 3.1% in Shanghai [11], 3.1% in Malawi [60], and 8.6% in Vietnam [61] but lower than Uzbekistan where the recurrence rate was 36% [62]. Recurrence rates are higher in high TB burden settings compared to European countries as represented by a recurrence rate of 0.66% in England and Wales [16] and 1.3% in Spain [63]. The higher recurrence rate we observed in our cohort might reflect the patient population attending the hospital although the prevalence of recurrent TB in this setting may be underestimated. This is because community-based TB diagnosis and treatment have not been fully captured in this cohort.

We established that older age is associated with recurrence. As immunity decreases with age and people with suppressed immunity are prone to infectious diseases and in a TB high burden setting chances of recurrence increase. A retrospective case-control study conducted in Singapore reported age ≥60 years to be related to TB recurrence [64] while a study from Pakistan reported a high recurrence rate in the younger age group (15–45 years) [25]. The difference between these reports likely reflects the difference in the etiology of recurrence. Singapore has a moderate TB incidence and TB elimination program has assured treatment completion rate. Therefore, recurrence is more likely to be due to reinfection. In Pakistan, relapse of disease arising from treatment failure is more likely because there is no tracking of treatment completion in Pakistan. The median age of patients with recurrent TB in our cohort was 32 years, consistent with the reported age range in Pakistan.

The association of sputum smear positivity with TB recurrence in our cohort reflects an increase in mycobacterial load, increasing the chance of recurrence as it is highly infectious. In a study conducted in California for investigating recurrence rate and risk factors associated with late recurrence, it was found that sputum smear-positive disease during primary TB is associated with late recurrence [17]. A patient-level pooled analysis of treatment-shortening regimens involving 3411 participants was conducted in 2018. The findings of the study showed that high smear grade resulted in treatment failure elevating the risk of recurrence [65]. In a study conducted in South Africa on 500 smear-positive pulmonary tuberculosis patients, 11% of the patients who successfully completed their treatment had TB recurrence. Recurrence in these patients is associated with high smear grade during the first episode of TB [66].

Drug resistance is another important factor associated with recurrent disease in the current study. We are unable to determine whether patients with drug-resistant recurrent TB in our study had this phenotype of disease at first presentation or acquired resistance at recurrence due to a lack of knowledge of drug resistance testing during first episode. A follow-up study in Henan province of China showed MDR-TB as a predictor for recurrence [22]. The odds of recurrence for MDR-TB patients reported from China are lower than in the present study and the reason might be a difference in the study design. A retrospective observational study from Uzbekistan also reported MDR-TB to be a risk factor for recurrence [62]. Both the abovementioned studies included participants who have successfully completed treatment. The higher odds of TB recurrence in the current study might be because of incomplete treatment and it is well-recognized that treatment failure carries an incremental risk of developing a drug-resistant disease.

Besides those sociodemographic and clinical factors included in this study, treatment failure due to inappropriate treatment is reported in 0.4%–45% of patients [67]. A study from India reported a 26% prevalence of inappropriate treatment [68] and another study from Benin reported a ≥10% prevalence in children [69]. Inappropriate treatment leading to treatment failure might be the reason for recurrence because of drugs with low bactericidal effects, inadequate treatment duration, and ignoring pre-existing drug resistance [70]. In Pakistan, a nine-month regimen including four drugs (isoniazid, rifampicin, ethambutol, and pyrazinamide) is recommended for treatment without testing for drug resistance. Still, medication use is not proper according to the physician's prescription. Although a positive response has been recorded for treatment completion by almost 62% of the patients in our cohort; however, this does not represent a true picture in all cases because patients comply in terms of getting medicines from healthcare centers but do not comply with using them. Though expensive, supervised treatment is a better option to ensure proper medication use and prevent illicit drug deviation [70].

A significant proportion of recurrent TB cases in our large case-control study is representative of the national picture. These cases are more often highly infectious leading to greater onward transmission, and a higher proportion are drug-resistant requiring longer and more complex treatment regimens to achieve cure. Strategies focused on improved management of patients presenting with TB as a first episode, including enhanced DOTS (directly observed treatment, short course) program, are likely to be cost-effective if they can succeed in improving treatment failure rates that predispose to recurrent TB.

### 4.1. Limitations of the Study

Our study is limited by the absence of information relating to previous TB treatment and outcomes in our cohort with a recurrent disease, which requires a longitudinal study. We also do not have data on the infecting strain at the times of initial and recurrent disease. We are therefore unable to distinguish between relapse and reinfection. Data on Xpert Mtb/RIF test for drug resistance are also missing during the first episode. Overcoming these shortcomings in clinical data integration for each incident TB episode and accessibility of tests for strain identification at each episode would empower a better study design in the future. Improved data infrastructure is needed to enhance the utility of surveillance studies like ours.

### 4.2. Conclusion

In this large case-control study, we sorted individuals with low BMI, having female gender, single/unmarried status, low socioeconomic status, being diabetic, and smokers to be at risk for developing TB. Among the cases, AFB sputum positivity, drug resistance, age, and awareness are identified as risk factors for TB recurrence. We recommend target screening based on the identified risk factors and further studies for investigating the time of recurrence and differentiating between relapse and reinfection.

## Figures and Tables

**Table 1:** Univariate analysis of sociodemographic and clinical characteristics of cases or controls (*n* = 3999) and multivariate model for TB cases.

Factors		*Descriptive statistics*		Univariate test^a^	*Multivariate test* ^*b*^	
		Controls (%)	Cases (%)	*χ* ^2^ (*p* value)	*p* value	Odds ratio (95% CI)
Age	Median (IQR)	35 (29)	30 (28)		0.461	1.001 (0.998–1.003)
BMI	Median (IQR)	20.7 (5.6)	18.5 (5.2)		<0.001	0.961 (0.954–0.968)
*Gender*	Females	14.1	39.6	329.46 (<0.001)	<0.001	2.065 (1.922–2.219)
	Males	85.9	60.4			

*Marital status*	Single	31.5	37.5	15.58 (<0.001)	0.003	1.214 (1.109–1.328)
	Married	68.5	62.5			

*Employment*	Unemployed	39.5	46.6	77.44 (<0.001)		
	Employed	60.5	53.4			

*Provincial distribution*	Punjabi	63.5	75.1	61.80 (<0.001)	<0.001	1.277 (1.189–1.373)
	Non-Punjabi	36.5	24.9			

*Socioeconomic status*	<25000	91.7	87.2	125.41 (<0.001)	0.937	0.995 (0.858–1.162)
	25001–40000	1.0	8.0		<0.001	1.935 (1.616–2.323)
	>40000	7.3	4.8			

*Educational status*	Higher secondary	31.2	33.2	10.03 (<0.001)	0.803	1.010 (0.934–1.093)
	Secondary	33.0	28.4		0.060	0.925 (0.853–1.003)
	Uneducated	35.8	38.4			

*BCG vaccinated*		22.6	27.3	11.74 (<0.001)		
*HIV positive*		0.1	0.25	1.50 (0.10)		
*Positive family history*		20.1	25	13.41 (<0.001)	0.239	1.044 (0.971–1.122)
*Smokers*		14.7	21.75	32.79 (<0.001)	<0.001	1.567 (1.435–1.710)
*Diabetes mellitus*		6.0	8.1	6.73 (<0.001)	0.023	1.142 (1.017–1.278)
*Cardiac vascular diseases*		99.1	1.8	0.63 (0.01)		

^a^ Pearson chi-square test was used for association of factors with cases and a *p* value of <0.05 was considered significant. ^b^ The multivariate regression model was used for the association of risk factors with cases. Only those factors were included in the model whose correlation coefficient is <0.6.

**Table 2:** Descriptive and univariate analyses of sociodemographic, clinical, and disease characteristics of TB patients stratified by first episode and recurrence and multivariate model for recurrent TB (*n* = 2000).

Factors		*Descriptive statistics*		Univariate test^a^	*Multivariate test * ^*b*^	
		Primary (%)	Recurrence (%)	*χ * ^*2*^ *p* value	*p* value	Odds ratio (95% CI)
Age	Median (IQR)	30 (28)	32 (23.5)	—	0.001	1.011 (1.004–1.017)
*BMI*	Median (IQR)	18.6 (5.2)	18.1 (4.2)	—	0.06	0.972 (0.942–1.001)
*Gender*	Females	39.5	40.4	0.03 (0.806)	—	—
	Males	60.5	59.6			

*Employment status*	Unemployed	53.9	49.3	1.49 (0.222)	—	—
	Employed	46.1	50.7			

*Provincial status*	Non-Punjabi	25.4	21.1	1.79 (0.182)	—	—
	Punjabi	74.6	78.9			

*Marital status*	Married	62.1	65.5	0.81 (0.369)	—	—
	Single	37.9	34.5			

*Socioeconomic status*	<25000	87.9	81.6	7.12 (0.028)	—	—
	25001–40000	7.7	11.2			
	>40000	4.4	7.2			

*Educational status*	Higher secondary	32.7	37.2	5.93 (0.052)	—	—
	Secondary	29.3	21.5			
	Uneducated	38.0	41.3			

*BCG vaccinated*		28.2	19.7	6.82 (0.009)	—	—
*HIV positive*		0.28	0	0.007 (0.935)	—	—
*Positive family history*		25.2	2.9	0.28 (0.594)	—	—
*Smokers*		21.3	3.2	1.45 (0.228)	—	—
*Diabetes mellitus*		8.3	6.7	0.44 (0.505)	—	—
*Cardiac vascular diseases*		1.7	2.7	0.63 (0.427)	—	—
*Type of TB*	EPTB ^*∗*^	16.8	9.0	8.44 (0.004)	—	—
	PTB ^*∗∗*^	83.2	91.0			

*Xpert MTB-RIF*	Tested	37.8	29.1	200.65 (<0.001)		
	Not tested	62.2	70.9			

*Awareness of TB*	No	78.7	60.1	37.14 (<0.001)		
	Yes	21.3	39.9		<0.001	1.906 (1.486–2.437)

*AFB sputum*	Negative	39.0	10.8	175.8 (<0.001)	0.03	0.580 (0.352–0.945)
	Nil	30.2	13.5			
	Positive	30.8	75.8		<0.001	2.384 (1.650–3.536)

*Drug resistance*	DS-TB ^*∗∗∗*^	98.1	61.0			
	DR-TB ^*∗∗∗∗*^	1.9	39.0	473.3 (<0.001)	<0.001	5.615 (4.265–7.386)

^a^ Pearson chi-square test was used for association of factors with cases and a *p* value of <0.05 was considered significant. ^b^ Multivariate regression model was used for the association of risk factors with cases. Only those factors were included in the model whose correlation coefficient is <0.6. *∗*extra-pulmonary tuberculosis,  ^*∗∗*^ pulmonary tuberculosis, ^∗∗∗^drug-sensitive tuberculosis, ^∗∗∗∗^drug-resistant tuberculosis.

## Data Availability

The data used to support the findings of this study are available from the corresponding author upon request.

## Supplementary Materials

Supplementary Materials Correlation coefficient values are presented in Table S1 and Table S2. Table S1: Correlation coefficient values showing the association of factors with one another for inclusion of independent factors in the multivariate model for TB cases. Table S2: Correlation coefficient values showing the association of factors with one another for inclusion of independent factors in the multivariate model for TB recurrence.
