# Supplementary material for: Demographic and Clinical Determinants of Tuberculosis and TB Recurrence: A Double-Edged Retrospective Study from Pakistan
Source: J Trop Med. 2022 Nov 28;2022:4408306. doi: 10.1155/2022/4408306 (PMC9722313; doi:10.1155/2022/4408306)
Supplement: Supplementary Materials — Correlation coefficient values are presented in Table S1 and Table S2. Table S1: Correlation coefficient values showing the association of factors with one another for inclusion of independent factors in the multivariate model for TB cases. Table S2: Correlation coefficient values showing the association of factors with one another for inclusion of independent factors in the multivariate model for TB recurrence. [file 4408306.f1.docx]

**Supplementary File**

**Title: Demographic and clinical determinants of tuberculosis and TB recurrence: a double-edged retrospective study from Pakistan**

Table S1: Correlation coefficient values showing the association of factors with one another for inclusion of independent factors in the multivariate model for TB cases.

| **Factors** | **Group** | **Gender** | **Employment Status** | **Province** | **Marital Status** | **Monthly Income** | **Education** | **BCG Vaccination** | **HIV Status** | **Family History** | **Smoking** | **Diabetes** | **Cardiac**  **Disease** |
| --- | --- | --- | --- | --- | --- | --- | --- | --- | --- | --- | --- | --- | --- |
| **Group** | 1 |  |  |  |  |  |  |  |  |  |  |  |  |
| **Gender** | 0.3 | 1.0 |  |  |  |  |  |  |  |  |  |  |  |
| **Employment Status** | 0.1 | 0.6 | 1.0 |  |  |  |  |  |  |  |  |  |  |
| **Province** | 0.1 | 0.0 | 0.1 | 1.0 |  |  |  |  |  |  |  |  |  |
| **Marital Status** | 0.1 | 0.0 | 0.4 | 0.1 | 1.0 |  |  |  |  |  |  |  |  |
| **Monthly Income** | 0.2 | 0.2 | 0.3 | 0.1 | 0.1 | 1.0 |  |  |  |  |  |  |  |
| **Education** | 0.1 | 0.2 | 0.0 | 0.1 | 0.3 | 0.1 | 1.0 |  |  |  |  |  |  |
| **BCG Vaccination** | 0.1 | 0.1 | 0.4 | 0.1 | 0.6 | 0.1 | 0.2 | 1.0 |  |  |  |  |  |
| **HIV Status** | 0.0 | 0.0 | 0.0 | 0.0 | 0.0 | 0.0 | 0.0 | 0.0 | 1.0 |  |  |  |  |
| **Family History** | 0.1 | 0.0 | 0.1 | 0.1 | 0.1 | 0.1 | 0.0 | 0.1 | 0.0 | 1.0 |  |  |  |
| **Smoking Cigarette** | 0.1 | 0.3 | 0.3 | 0.0 | 0.2 | 0.2 | 0.1 | 0.2 | 0.0 | 0.0 | 1.0 |  |  |
| **Diabetes** | 0.0 | 0.0 | 0.0 | 0.0 | 0.1 | 0.0 | 0.0 | 0.1 | 0.0 | 0.0 | 0.1 | 1.0 |  |
| **Cardiac Disease** | 0.0 | 0.0 | 0.0 | 0.0 | 0.0 | 0.0 | 0.0 | 0.0 | 0.0 | 0.0 | 0.0 | 0.0 | 1.0 |

Table S2: Correlation coefficient values showing the association of factors with one another for inclusion of independent factors in the multivariate model for TB recurrence.

| **Factors** | **Stage of TB** | **Gender** | **Employment Status** | **Province** | **Marital Status** | **Monthly Income** | **Education** | **BCG Vaccination** | **HIV Status** | **Family History** | **Diabetes** | **Cardiac Disease** | **Smoking** | **Type of TB** | **Awareness** | **XPERT MTB** | **AFB Sputum** | **DR** |
| --- | --- | --- | --- | --- | --- | --- | --- | --- | --- | --- | --- | --- | --- | --- | --- | --- | --- | --- |
| **Stage of TB** | 1.0 |  |  |  |  |  |  |  |  |  |  |  |  |  |  |  |  |  |
| **Gender** | 0.0 | 1.0 |  |  |  |  |  |  |  |  |  |  |  |  |  |  |  |  |
| **Employment Status** | 0.0 | 0.7 | 1.0 |  |  |  |  |  |  |  |  |  |  |  |  |  |  |  |
| **Province** | 0.0 | 0.0 | 0.1 | 1.0 |  |  |  |  |  |  |  |  |  |  |  |  |  |  |
| **Marital Status** | 0.0 | 0.0 | 0.2 | 0.1 | 1.0 |  |  |  |  |  |  |  |  |  |  |  |  |  |
| **Monthly Income** | 0.1 | 0.3 | 0.4 | 0.1 | 0.1 | 1.0 |  |  |  |  |  |  |  |  |  |  |  |  |
| **Education** | 0.1 | 0.2 | 0.1 | 0.1 | 0.3 | 0.1 | 1.0 |  |  |  |  |  |  |  |  |  |  |  |
| **BCG Vaccination** | 0.1 | 0.1 | 0.3 | 0.1 | 0.6 | 0.1 | 0.2 | 1.0 |  |  |  |  |  |  |  |  |  |  |
| **HIV Status** | 0.0 | 0.0 | 0.1 | 0.0 | 0.0 | 0.0 | 0.0 | 0.0 | 1.0 |  |  |  |  |  |  |  |  |  |
| **Family History** | 0.0 | 0.0 | 0.0 | 0.1 | 0.2 | 0.1 | 0.0 | 0.2 | 0.0 | 1.0 |  |  |  |  |  |  |  |  |
| **Diabetes** | 0.0 | 0.0 | 0.0 | 0.0 | 0.0 | 0.0 | 0.0 | 0.1 | 0.1 | 0.0 | 1.0 |  |  |  |  |  |  |  |
| **Cardiac Disease** | 0.0 | 0.0 | 0.0 | 0.0 | 0.0 | 0.0 | 0.0 | 0.0 | 0.0 | 0.0 | 0.0 | 1.0 |  |  |  |  |  |  |
| **Smoking** | 0.0 | 0.4 | 0.4 | 0.0 | 0.2 | 0.2 | 0.1 | 0.2 | 0.1 | 0.0 | 0.0 | 0.0 | 1.0 |  |  |  |  |  |
| **Type of TB** | 0.1 | 0.0 | 0.0 | 0.0 | 0.0 | 0.0 | 0.1 | 0.0 | 0.0 | 0.1 | 0.0 | 0.0 | 0.0 | 1.0 |  |  |  |  |
| **Awareness** | 0.1 | 0.1 | 0.0 | 0.0 | 0.1 | 0.1 | 0.1 | 0.1 | 0.0 | 0.6 | 0.0 | 0.0 | 0.0 | 0.0 | 1.0 |  |  |  |
| **XPERT MTB** | 0.3 | 0.0 | 0.0 | 0.0 | 0.0 | 0.0 | 0.0 | 0.1 | 0.0 | 0.0 | 0.0 | 0.0 | 0.1 | 0.3 | 0.0 | 1.0 |  |  |
| **AFB Sputum** | 0.3 | 0.0 | 0.1 | 0.1 | 0.1 | 0.0 | 0.1 | 0.1 | 0.0 | 0.0 | 0.0 | 0.0 | 0.1 | 0.6 | 0.1 | 1.0 | 1.0 |  |
| **DR** | 0.5 | 0.1 | 0.1 | 0.0 | 0.0 | 0.1 | 0.0 | 0.0 | 0.0 | 0.0 | 0.1 | 0.0 | 0.0 | 0.1 | 0.0 | 0.3 | 0.3 | 1.0 |
